# Antibody-drug conjugate combinations in cancer treatment: clinical efficacy and clinical study perspectives

**DOI:** 10.3389/fphar.2025.1556245

**Published:** 2025-02-21

**Authors:** Xianglong Shi, Kai Tang, Quanbin Zhang, Qingkun Han, Lin Quan, Yijing Li, Jianqiao Cui, Nuan Feng, Jianbao Gong, Baoxin Shang, Xuwen Li

**Affiliations:** ^1^ The Afffliated Hospital of Qingdao University, Qingdao University, Qingdao, China; ^2^ Department of Urology, Zibo Central Hospital, Zibo, China; ^3^ Department of Trauma Orthopedics, Zibo Central Hospital, Zibo, China; ^4^ Hematology Department, Zibo Central Hospital, Zibo, China; ^5^ Qingdao Women and Children’s Hospital, Qingdao University, Qingdao, China; ^6^ Qingdao Hospital, University of Health and Rehabilitation Sciences, Qingdao Municipal Hospital, Qingdao, China; ^7^ Traumatology Department, Affiliated Hospital of Qingdao University, Qingdao, China

**Keywords:** antibody-drug conjugates (ADC), cancer therapy, combination strategies, clinical studies, tumor microenvironment

## Abstract

Antibody-drug conjugates have emerged as a promising cancer treatment, combining targeted delivery of cytotoxic agents with the specificity of monoclonal antibodies. Despite their potential, ADCs face limitations such as resistance and off-target effects. To enhance their efficacy, ADCs are increasingly being combined with other therapeutic strategies, including immune checkpoint inhibitors, chemotherapy, small-molecule inhibitors, anti-angiogenic agents, and CAR-T cell therapies. These combination therapies aim to overcome resistance mechanisms, improve tumor targeting, and boost immune responses. Clinical studies have shown that such combinations can significantly improve response rates and progression-free survival across various cancers. This review explores the mechanisms, clinical efficacy, key studies, challenges, and future perspectives of Antibody-drug conjugates combinations in cancer therapy.

## 1 Introduction

Cancer remains one of the leading causes of death worldwide, with millions of new diagnoses each year. Despite significant advances in cancer treatment, the prognosis for patients with advanced and metastatic cancers remains poor, particularly for malignancies that are resistant to conventional therapies such as chemotherapy and radiation (Goswami et al., 2024; Zhang and Wu, 2023; Misawa et al., 2025; Xu W. et al., 2024; Zhang et al., 2023). In recent years, ADCs have emerged as a promising therapeutic strategy, offering targeted drug delivery to cancer cells while minimizing off-target toxicity (Dumontet et al., 2023; Liu et al., 2023). ADCs consist of a monoclonal antibody linked to a potent cytotoxic drug, allowing for precise targeting of tumor cells based on specific surface antigens, such as HER2, CD20, and Trop-2 (Shih et al., 2024; Belluomini et al., 2023a).

While ADCs have demonstrated significant efficacy in certain cancers, their full potential is often limited by factors such as resistance mechanisms, side effects, and the complexity of tumor biology (Díaz-Rodríguez et al., 2022; Tsuchikama et al., 2024; Zhao et al., 2023). To overcome these limitations, researchers have increasingly explored the potential of combining ADCs with other therapeutic modalities, including immune checkpoint inhibitors, small-molecule targeted therapies, and traditional chemotherapies (Wei et al., 2024; Nicolò et al., 2022). The rationale behind these combination therapies is to enhance the overall therapeutic effect by attacking tumors through multiple mechanisms, thereby overcoming resistance and improving clinical outcomes (Lu et al., 2023; Yu et al., 2024; Jiang et al., 2024).

Combination therapies leveraging ADCs have shown promise across a variety of cancers, including breast cancer, lung cancer, urothelial carcinoma, and lymphoma (Wei et al., 2024). In these settings, ADCs work synergistically with immune modulators and chemotherapy agents to not only target and destroy tumor cells but also to stimulate the immune system, enhance tumor-specific responses, and promote long-term remission. This combination approach represents a new Frontier in cancer therapy, with the potential to significantly improve patient outcomes, reduce side effects, and ultimately change the treatment paradigm for several difficult-to-treat cancers (Wang et al., 2022).

## 2 Mechanisms of action of ADCs and their combinations

ADCs are designed to deliver potent cytotoxic drugs directly to cancer cells while sparing normal tissues. The core mechanism of ADCs relies on the specificity of monoclonal antibodies (mAbs) that target tumor-associated antigens, such as HER2, CD20, and Trop-2 (Filis et al., 2023). However, the effectiveness of ADCs is often constrained by several factors, including antigen heterogeneity, resistance mechanisms, and off-target toxicity (Parakh et al., 2021). Antigen heterogeneity, such as varying levels of HER2 expression in breast cancer, can result in suboptimal binding and reduced drug delivery, while changes in antigen expression during disease progression further diminish ADC efficacy (Schettini and Prat, 2021; Li et al., 2024). Resistance mechanisms include antigen downregulation, as seen in Trop-2-targeting ADCs like Sacituzumab govitecan, overexpression of multidrug resistance transporters like P-glycoprotein that expel cytotoxic payloads, and impaired lysosomal function that hinders payload release (Abelman et al., 2023; Chen et al., 2023). Additionally, off-target toxicity remains a challenge, with unintended binding and payload leakage contributing to adverse effects. For instance, Enfortumab vedotin, targeting Nectin-4, has been linked to peripheral neuropathy, and unstable linkers in earlier ADCs have caused systemic toxicity. Addressing these limitations is crucial for improving ADC therapeutic outcomes (Aoyama et al., 2022; Sardinha et al., 2023).

To overcome these challenges, ADCs are increasingly being combined with other therapeutic strategies to enhance their efficacy. One such combination is with immune checkpoint inhibitors, like anti-PD-1 or anti-CTLA-4 antibodies. These inhibitors work by reactivating the immune system to recognize and destroy cancer cells. When combined with ADCs, immune checkpoint inhibitors can help eliminate tumors that evade immune detection, thereby enhancing both the immune response and the cytotoxic effects of ADCs (Wang et al., 2023).

**FIGURE 1:**
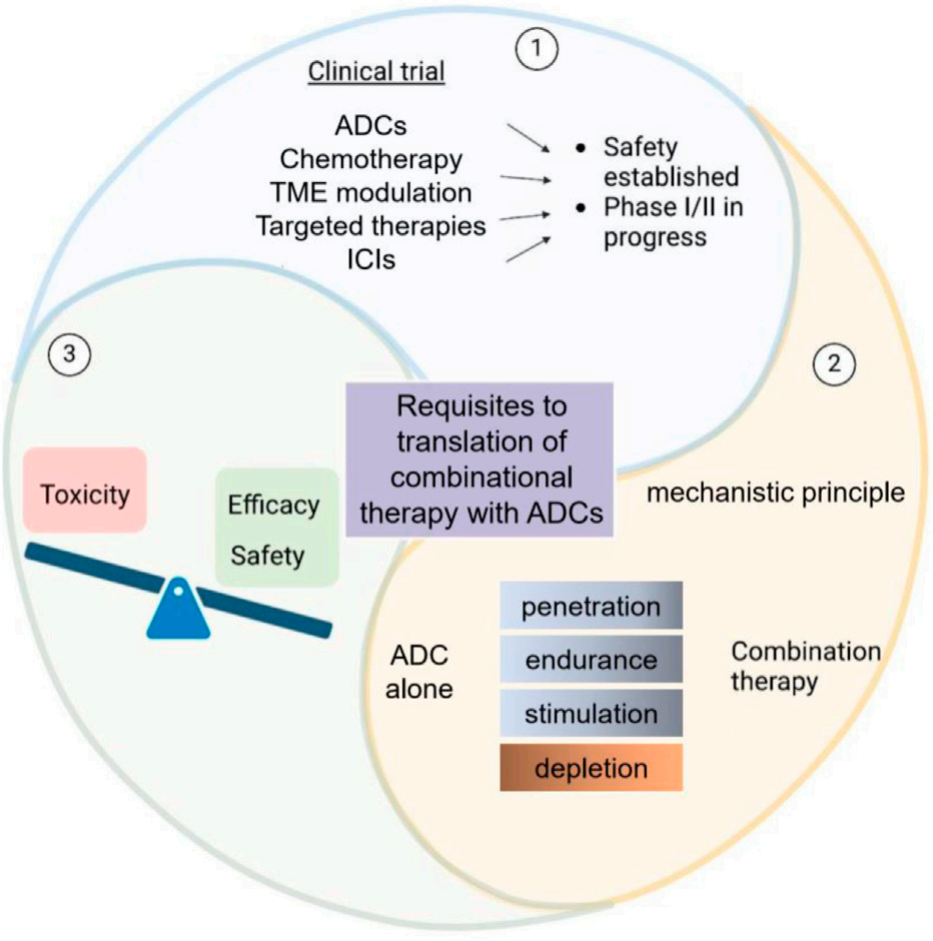
Schematic overview of ADCs in clinical trials and combinational therapy requirements. This diagram depicts the considerations in ADC-based clinical trials. It balances toxicity, efficacy, and safety for ADCs alone. Additionally, it outlines the prerequisites for translating ADC - based combinational therapy, featuring mechanistic principles and therapy components such as penetration and depletion.

In addition, ADCs are being combined with targeted therapies, such as small-molecule inhibitors of tyrosine kinases, which can disrupt key signaling pathways in tumor cells. These combinations can prevent the activation of compensatory survival mechanisms, increasing the likelihood that the ADC will successfully kill the tumor cell (Abuhelwa et al., 2022). Chemotherapy can also be combined with ADCs to enhance cytotoxicity through synergistic effects (Wei et al., 2024). By attacking the tumor from multiple angles, these combination therapies hold the potential to overcome resistance and improve patient outcomes in cancers that are difficult to treat with monotherapy (Figure 1).

## 3 Current ADC options in clinical practice

ADCs are complex molecules composed of three key components: (Goswami et al., 2024): a monoclonal antibody that specifically binds to a tumor-associated antigen, (Zhang and Wu, 2023), a cytotoxic payload that kills cancer cells, and (Misawa et al., 2025) a chemical linker that connects the antibody to the payload. The antibody ensures targeted delivery of the payload to cancer cells, while the linker controls the release of the payload, minimizing off-target toxicity (Katrini et al., 2024). Several ADCs have been approved for clinical use, each with unique structural and functional characteristics. For example, Trastuzumab emtansine (T-DM1) consists of the HER2-targeting antibody trastuzumab linked to the maytansinoid derivative DM1 via a non-cleavable thioether linker. Upon internalization, DM1 is released and disrupts microtubule assembly, leading to cell cycle arrest and apoptosis in HER2-positive breast cancer cells (Barok et al., 2014). Similarly, Enfortumab vedotin combines an anti-Nectin-4 antibody with the microtubule-disrupting agent monomethyl auristatin E (MMAE) through a cleavable protease-sensitive linker. This ADC is approved for advanced urothelial carcinoma and demonstrates potent tumor-killing activity (Challita-Eid et al., 2016). Another example, Sacituzumab govitecan, targets Trop-2 using a humanized antibody linked to SN-38, a topoisomerase I inhibitor, via a hydrolyzable linker. This ADC has shown significant efficacy in triple-negative breast cancer (TNBC) by inducing DNA damage and cell death (Bardia et al., 2021). These ADCs exemplify the potential of targeted drug delivery, but challenges such as resistance mechanisms and off-target effects persist, underscoring the need for innovative combination strategies to further enhance their therapeutic impact.

## 4 Clinical efficacy and advances in ADC combinations

ADCs have revolutionized targeted cancer therapies by combining the specificity of monoclonal antibodies with the potent cytotoxic effects of chemotherapeutics. However, the clinical success of ADCs often requires rationally designed combination therapies to overcome limitations such as resistance, antigen heterogeneity, and tumor microenvironment barriers (Wei et al., 2024). This section explores the efficacy of ADC combinations, key clinical trials, and their implications for future therapeutic strategies. For clarity, the discussion is divided into two parts: Section 4.1 focuses on the clinical outcomes of ADC combinations, while Section 4.2 highlights pivotal trials and ongoing studies that have advanced this field.

### 4.1 Clinical efficacy of ADC combinations

The combination of ADCs with other therapeutic modalities has shown immense promise in improving the efficacy of cancer treatments. ADCs, designed to deliver cytotoxic agents directly to tumor cells via targeted antibodies, can be further optimized by combining them with chemotherapy, immunotherapy, and other targeted agents. These combinations have demonstrated substantial improvements in clinical outcomes, including overall survival (OS), progression-free survival (PFS), and overall response rates (ORR), and are a key focus in cancer therapeutics (Kang et al., 2024).

#### 4.1.1 ADCs and chemotherapy

In clinical trials, resistance to ADCs has been reported as a significant barrier to durable responses (Belluomini et al., 2023b). For instance, in HER2-positive breast cancer, resistance to T-DM1 has been associated with HER2 downregulation and upregulation of compensatory signaling pathways such as PI3K/AKT (Dey et al., 2024). Similarly, resistance to Trop-2-targeted Sacituecan in TNBC has been linked to increased drug efflux activity mediated by MDR transporters. Combining ADCs with traditional chemotherapeutic agents aims to exploit their synergistic effects, overcoming tumor resistance mechanisms and enhancing treatment efficacy.

##### 4.1.1.1 Combination of ADCs and taxanes

Taxanes, such as Docetaxel and Paclitaxel, are widely used in various cancers, including breast cancer, lung cancer, and ovarian cancer (Sun et al., 2022; Mosca et al., 2021). These drugs work by stabilizing microtubules, preventing cell division, and ultimately leading to cell death. The combination of taxanes with ADCs, particularly in HER2-positive breast cancer, has shown promising results.

For instance, the combination of T-DM1 with Paclitaxel has been explored in HER2-positive breast cancer (Ruddy et al., 2021). Clinical trials have demonstrated that this combination leads to enhanced tumor regression, improved PFS, and a reduction in the incidence of disease progression compared to monotherapy with either agent alone. This combination benefits from the synergistic effects of Paclitaxel, which may enhance ADC internalization and drug delivery (Krop et al., 2016).

##### 4.1.1.2 Combination of ADCs and platinum-based chemotherapies

Platinum-based agents like Cisplatin and Carboplatin are frequently used in solid tumors such as ovarian, non-small cell lung cancer (NSCLC), and head and neck cancers (Zhang et al., 2022). ADCs targeting tumor-specific antigens, such as Enfortumab vedotin (anti-Nectin-4) and Sacituzumab govitecan (anti-Trop-2), are being combined with platinum-based chemotherapies to enhance their cytotoxic effect (Belluomini et al., 2023a; Wong and Rosenberg, 2021). This approach may reduce the development of resistance and improve the distribution of ADCs within the tumor by inducing DNA damage and enhancing tumor cell death.

For example, Sacituzumab govitecan combined with Cisplatin in TNBC has shown to significantly improve ORR and PFS in clinical trials, providing a promising strategy for patients with metastatic or resistant TNBC (Tagawa et al., 2024).

#### 4.1.2 ADCs and immune checkpoint inhibitors

Immunotherapy, particularly immune checkpoint inhibitors (ICIs) targeting PD-1/PD-L1 or CTLA-4, has significantly improved cancer treatment outcomes (Wang et al., 2024). Combining ADCs with ICIs aims to harness the power of the immune system to eradicate cancer cells while maintaining the targeted cytotoxic action of ADCs.

##### 4.1.2.1 Combination of ADCs and PD-1/PD-L1 inhibitors

The combination of ADCs with PD-1/PD-L1 inhibitors is an active area of clinical investigation. Atezolizumab (anti-PD-L1) and Pembrolizumab (anti-PD-1) have been combined with ADCs such as Enfortumab vedotin (anti-Nectin-4) in urothelial carcinoma (Nucera et al., 2024). In clinical trials, these combinations have resulted in significantly improved ORR, OS, and PFS compared to monotherapy. The rationale behind these combinations is that PD-1/PD-L1 blockade enhances T-cell activation and immune responses, allowing the immune system to more effectively recognize and eliminate tumor cells, including those targeted by ADCs (Pessino et al., 2024).

In HER2-positive breast cancer, T-DM1 combined with Pembrolizumab has shown encouraging early-phase clinical results (Waks et al., 2022). The addition of the immune checkpoint inhibitor boosts the immune response against residual tumor cells, which is particularly important in metastatic settings where immune evasion is a major challenge (Waks et al., 2024).

##### 4.1.2.2 Combination of ADCs and CTLA-4 inhibitors

The combination of T-DM1 with Ipilimumab (anti-CTLA-4) is being explored in clinical trials for HER2-positive and other solid tumors (Müller et al., 2015). CTLA-4 inhibitors stimulate T-cell activation and can help overcome immune suppression within the TME. Combining Ipilimumab with ADCs may synergize by improving immune-mediated destruction of cancer cells while the ADC delivers targeted cytotoxicity directly to tumor cells. Early results suggest that this combination may improve survival rates and reduce disease progression in patients with metastatic cancer.

#### 4.1.3 ADCs and targeted therapies

Targeted therapies that block specific molecular pathways involved in cancer growth can be combined with ADCs to provide a multi-pronged approach to cancer treatment (Pérez-Herrero and Fernández-Medarde, 2015; Gujarathi et al., 2024). These combinations aim to target both the tumor and its supporting environment, leading to enhanced tumor regression and reduced relapse rates.

##### 4.1.3.1 Combination of ADCs and tyrosine kinase inhibitors (TKIs)

TKIs, such as Lapatinib (targeting HER2 and EGFR) and Osimertinib (targeting EGFR), are used in cancers with specific mutations. Combining these inhibitors with ADCs like T-DM1 has been studied in HER2-positive breast cancer (Scheck et al., 2024; Aggarwal et al., 2023). Lapatinib enhances the therapeutic effects of T-DM1 by blocking signaling pathways that promote tumor cell survival, thus improving ADC efficacy. The combination has shown promising results in metastatic breast cancer, particularly in patients who have developed resistance to single-agent therapies.

##### 4.1.3.2 Combination of ADCs and anti-angiogenesis agents

Bevacizumab, an anti-VEGF monoclonal antibody, is used to block angiogenesis, thereby limiting the tumor’s ability to grow new blood vessels. When combined with ADCs such as T-DM1, Bevacizumab helps improve ADC penetration into solid tumors by reducing the dense extracellular matrix and normalizing the blood vessel structure within tumors. In clinical studies, this combination has shown increased tumor shrinkage and improved clinical outcomes in patients with HER2-positive breast cancer (Pondé et al., 2020).

#### 4.1.4 ADCs and tumor microenvironment modulation

The tumor microenvironment (TME), characterized by fibrosis, hypoxia, and immunosuppressive factors, can create barriers to effective drug delivery and reduce treatment efficacy. Combining ADCs with therapies that modulate the TME is a promising strategy for improving treatment outcomes. The dense stromal components of the TME can impede the penetration of ADCs into tumors. Interleukin-2 (IL-2) and other immune modulators can be combined with ADCs to promote T-cell activation and overcome immunosuppressive barriers in the TME. For instance, T-DM1 combined with IL-2 or GM-CSF (Granulocyte-Macrophage Colony-Stimulating Factor) is being explored to enhance immune responses, leading to better control of residual disease and prolonged survival (Xu M. et al., 2024).

In summary, the clinical efficacy of ADC combinations in cancer therapy has made significant strides, providing a multi-faceted approach to overcoming resistance and improving therapeutic outcomes. Combining ADCs with chemotherapy, immunotherapy, targeted therapies, and tumor microenvironment modulators not only enhances tumor-specific drug delivery but also works synergistically to improve overall treatment responses. These combinations have shown promise in multiple cancers, including breast cancer, urothelial carcinoma, leukemia, and solid tumors like pancreatic cancer. While the combination of therapies continues to evolve, the next-generation of ADC combinations is likely to further define cancer treatment paradigms, offering more personalized and effective treatment options for patients. With ongoing clinical trials and refinement of treatment regimens, ADC-based combination therapies are poised to play a critical role in advancing cancer treatment.

### 4.2 Key clinical studies and ongoing trials

The development of ADCs in combination therapies has shown significant promise in a variety of cancer types, highlighting their potential to improve patient outcomes. Numerous clinical studies and ongoing trials have been pivotal in advancing our understanding of the clinical efficacy and safety of ADC combinations. These trials span multiple malignancies, including breast cancer, urothelial carcinoma, lymphoma, and others, and have underscored the advantages of combining ADCs with other therapeutic modalities, such as immune checkpoint inhibitors, targeted therapies, and traditional chemotherapy.

#### 4.2.1 Trastuzumab emtansine (T-DM1) and Pembrolizumab in HER2-Positive breast cancer

One of the key studies in HER2-positive metastatic breast cancer (MBC) is the Phase II trial combining Trastuzumab emtansine (T-DM1), an ADC targeting HER2, with Pembrolizumab, an anti-PD-1 immune checkpoint inhibitor (Janjigian et al., 2023). This study aimed to investigate whether the combination could enhance immune responses while also leveraging the cytotoxic properties of T-DM1. Early results from this trial indicated improved progression-free survival (PFS) and overall survival (OS) in patients who had previously been heavily treated. The synergy between these two agents appears to work by enhancing the immune system’s ability to recognize and attack tumor cells, while T-DM1 directly delivers its chemotherapeutic payload to the cancer cells. Notably, the combination was well tolerated, with manageable side effects, mainly related to Pembrolizumab, such as fatigue and immune-related adverse events. These findings suggest that ADCs can work synergistically with immune checkpoint inhibitors, potentially offering a novel treatment strategy for HER2-positive breast cancer (Waks et al., 2022; Smyth and Sundar, 2023).

#### 4.2.2 Sacituzumab govitecan and Pembrolizumab in triple-negative breast cancer (TNBC)

In a Phase I/II clinical trial exploring Sacituzumab govitecan, an ADC targeting Trop-2, in combination with Pembrolizumab, an anti-PD-1 antibody, the combination therapy has shown promising results for patients with metastatic triple-negative breast cancer (TNBC) (Grivas et al., 2024). TNBC is notoriously difficult to treat due to the lack of targeted therapies, and patients often face poor prognosis. This trial demonstrated that combining Sacituzumab govitecan with Pembrolizumab resulted in a significantly higher overall response rate (ORR) compared to monotherapies. Patients in this study also exhibited durable responses, with some experiencing long-term remission. The safety profile was manageable, with adverse effects such as diarrhea and neutropenia primarily linked to Sacituzumab govitecan. The success of this combination suggests that combining ADCs with immunotherapy could provide a potent treatment for TNBC, which remains a major unmet clinical need (Tolaney et al., 2024).

#### 4.2.3 Enfortumab vedotin and Pembrolizumab in urothelial carcinoma

The combination of Enfortumab vedotin, an ADC targeting Nectin-4, with Pembrolizumab is currently being evaluated in patients with metastatic urothelial carcinoma (UC) (Niegisch, 2024; Powles et al., 2024). UC, particularly in its advanced stages, is a difficult-to-treat cancer with few effective therapies. A Phase II study demonstrated that this combination resulted in substantial improvements in both progression-free survival (PFS) and overall response rates (ORR) in patients who had previously failed platinum-based chemotherapy. The combination works by targeting the cancer cells with the cytotoxic payload of Enfortumab vedotin while also stimulating the immune system through Pembrolizumab. Early results indicate a favorable safety profile, with manageable side effects including rash, fatigue, and peripheral neuropathy. This study underscores the potential for ADC-immunotherapy combinations to offer a new therapeutic approach for patients with advanced urothelial carcinoma (Hoimes et al., 2022; Bantounou et al., 2023; O'Donnell et al., 2023).

#### 4.2.4 Ongoing trials and future directions

Beyond the completed and ongoing studies mentioned above, many other clinical trials are investigating the potential of ADC combinations in various cancer types. These trials are exploring combinations with other targeted therapies, small molecule inhibitors, and chemotherapy agents to further enhance ADC efficacy. For example, combinations of ADCs with TKIs are being tested in lung cancer, while others are evaluating ADCs combined with anti-angiogenic agents to target the tumor vasculature.

In the hematological malignancies space, several trials are investigating the combination of ADCs with immunotherapies or other targeted therapies. One example is the combination of Polatuzumab vedotin, an anti-CD79 b ADC, with Rituximab (a CD20-targeting monoclonal antibody) in patients with relapsed or refractory non-Hodgkin lymphoma (NHL). Early results have shown promising efficacy, with many patients achieving complete responses. These ongoing studies are crucial in determining the full potential of ADC combinations in treating hematological cancers (Vodicka et al., 2022; Morschhauser et al., 2019; Terui et al., 2021).

Moreover, ongoing trials are exploring combinations with other immune checkpoint inhibitors, such as CTLA-4 inhibitors, to further enhance the anti-tumor immune response and overcome resistance mechanisms in tumors that evade immune detection. By combining ADCs with agents that modulate immune checkpoints or other immunological pathways, these trials aim to improve the overall efficacy of cancer therapies, especially in tumors with complex immune evasion mechanisms (Müller et al., 2015).

In summary, these key clinical studies demonstrate the promise of ADC combinations in improving clinical outcomes across a variety of cancer types (Table 1). As these studies progress into later stages of development, the combination of ADCs with immunotherapy, targeted therapies, and chemotherapy is expected to become an increasingly important strategy in cancer treatment. The results so far have been encouraging, and these therapies hold the potential to significantly enhance the treatment landscape for patients with cancers that are difficult to treat with conventional therapies alone.

**TABLE 1:** Summary of combination therapies involving ADCs in cancer treatment.

Combination therapy	Therapeutic agents	Mechanism of action	Targeted cancer types	Key findings
ADCs + Immune Checkpoint Inhibitors	- PD-1 inhibitors (e.g., Pembrolizumab)	- ADCs deliver cytotoxic drugs to tumor cells; immune checkpoint inhibitors reactivate immune response against cancer cells	HER2-positive breast cancer, triple-negative breast cancer, urothelial carcinoma	- ADCs enhance immune responses by reactivating immune surveillance - Combined therapy increases progression-free survival. - Can improve tumor-specific responses
	- PD-L1 inhibitors (e.g., Atezolizumab)	- Block PD-1/PD-L1 interactions to enhance T-cell activity against cancer cells		- Synergistic effects observed in improving overall response rates in various cancers - Immune-related adverse events like fatigue and rash are manageable
ADCs + Chemotherapy	- Traditional chemotherapies (e.g., paclitaxel, carboplatin)	- Chemotherapy enhances ADC efficacy by directly killing proliferating tumor cells while ADCs target specific antigens	Non-small cell lung cancer, breast cancer, lymphoma	- Increased tumor response when ADCs and chemotherapy are used together - Synergistic cytotoxic effects - Risk of overlapping toxicities like neutropenia and GI symptoms
	- Taxanes (e.g., Docetaxel)	- ADCs kill tumor cells while chemotherapy accelerates tumor cell death and inhibits growth		- Enhanced tumor control, particularly in chemotherapy-resistant cancers - Potential for reduced drug resistance when used in combination
ADCs + Targeted Small-Molecule Inhibitors	- Tyrosine kinase inhibitors (e.g., Erlotinib, Lapatinib)	- ADCs target cancer-specific antigens, while TKIs inhibit downstream signaling pathways critical for cancer cell survival	Lung cancer, breast cancer, other solid tumors	- ADCs combined with TKIs enhance anti-cancer effects and block compensatory signaling - Can improve outcomes in cancers with resistance to monotherapy
	- AKT inhibitors (e.g., Ipatasertib)	- ADCs deliver cytotoxic agents, while AKT inhibitors block cell survival pathways triggered by PI3K/AKT/mTOR signaling		- Synergistic cytotoxicity observed, especially in breast cancer and other solid tumors - Potential to overcome resistance by blocking adaptive survival pathways
ADCs + Anti-Angiogenic Agents	- VEGF inhibitors (e.g., Bevacizumab)	- ADCs target tumor cells, while anti-angiogenics inhibit vascular growth, improving drug delivery and reducing tumor hypoxia	HER2-positive breast cancer, ovarian cancer	- Anti-angiogenic agents increase ADC penetration in tumors by normalizing the vasculature - Improved progression-free survival and enhanced response rates in clinical trials
	- VEGFR inhibitors (e.g., Axitinib)	- Disrupts blood vessel formation within tumors, increasing ADC delivery		- Tumor vascular normalization improves ADC efficacy - Decreased drug resistance due to better tumor perfusion
ADCs + CAR-T Cell Therapy	- Chimeric Antigen Receptor T-cell (CAR-T) therapy	- ADCs deliver targeted cytotoxic agents, while CAR-T cells kill cancer cells through engineered immune responses	Hematological malignancies, solid tumors	- Synergistic effect observed in hematologic cancers like leukemia and lymphoma - CAR-T cells help further target antigen-positive tumor cells after ADC delivery
	- CAR-T (e.g., Kymriah, Yescarta)	- Combining CAR-T’s targeting capabilities with ADC’s cytotoxic payload enhances tumor cell targeting and immune destruction		- Early-phase studies show promising results - Challenges remain in optimizing dosing and reducing CAR-T related toxicity
ADCs + Immune Modulators	- TLR agonists (e.g., CpG oligodeoxynucleotides)	- ADCs deliver targeted toxins while TLR agonists activate innate immune responses to boost adaptive immunity	Melanoma, breast cancer, ovarian cancer	- TLR agonists enhance the immune response induced by ADCs - Increases tumor-specific immunity and reduces tumor growth
	- STING agonists (e.g., ADU-S100)	- Activates the STING pathway to induce tumor cell death and promote immune response, complementing ADC cytotoxicity	Various cancers (e.g., melanoma, breast cancer)	- Synergistic tumor destruction through enhanced immune activation - Significant increase in tumor regression in preclinical models
ADCs + Other Immunotherapies	- CTLA-4 inhibitors (e.g., Ipilimumab)	- ADCs deliver cytotoxic drugs to tumor cells while CTLA-4 inhibitors enhance T-cell-mediated immune responses	Melanoma, non-small cell lung cancer, head and neck cancers	- Combining ADCs with CTLA-4 inhibitors increases T-cell activation and tumor rejection - Promising results in early-phase trials for advanced cancers
	- OX40 agonists (e.g., MedImmune’s MEDI-575)	- ADCs and OX40 agonists work together to enhance anti-tumor T-cell responses and cytotoxic activity	Solid tumors, including melanoma and breast cancer	- Synergistic T-cell activation and increased anti-tumor activity - Tumor-specific immunity improves while reducing immune evasion

## 5 Concluding insights: overcoming challenges and shaping the future of ADC combinations

The clinical development of ADCs in combination with other therapeutic modalities has demonstrated substantial promise in advancing cancer treatment. Combining ADCs with immune checkpoint inhibitors, targeted therapies, and chemotherapy has yielded encouraging clinical results across a range of cancers, including HER2-positive breast cancer, triple-negative breast cancer, urothelial carcinoma, and lymphoma. The synergy between ADCs and other therapies enhances therapeutic efficacy by leveraging multiple mechanisms of action, including direct cytotoxicity, immune modulation, and targeted tumor destruction. These combination strategies not only improve treatment outcomes but also offer hope for patients with cancers that have limited therapeutic options.

However, despite these promising developments, several challenges remain. One significant hurdle is the management of adverse effects, which can become more pronounced when combining ADCs with other treatment regimens. While ADCs typically have more targeted effects, the combination with chemotherapy or immune checkpoint inhibitors can lead to overlapping toxicities, such as neutropenia, diarrhea, fatigue, and immune-related adverse events. Careful dose optimization and monitoring are essential to minimize these toxicities and improve the overall safety profile of combination therapies. Moreover, the development of resistance to ADCs and other therapeutic agents remains a persistent issue. Tumors may evolve mechanisms to evade the effects of ADCs, such as altering drug target expression or activating compensatory signaling pathways. Overcoming these resistance mechanisms will require more in-depth understanding of tumor biology and the molecular factors that drive treatment failure.

In the realm of immuno-oncology, combining ADCs with next-generation immune checkpoint inhibitors, such as those targeting LAG-3, TIM-3, and TIGIT, holds the potential to further enhance immune responses and overcome immune evasion. Additionally, the combination of ADCs with cellular therapies, such as CAR-T cells, represents an exciting avenue for exploration. These strategies could potentially provide a multi-pronged approach to fighting cancer by utilizing both direct cytotoxicity and immune-based tumor destruction. In conclusion, while ADC combinations have already shown substantial promise in clinical settings, there are still many challenges to overcome, particularly in terms of safety, resistance mechanisms, and patient stratification. Continued research and clinical trials will be essential in addressing these issues and unlocking the full potential of ADC combination therapies. With ongoing advancements in technology, biomarker discovery, and molecular engineering, ADCs are poised to play a central role in the future of cancer therapy, offering more personalized, effective, and less toxic treatment options for patients worldwide.

Overcoming these limitations through rationally designed combination therapies is critical. For example, combining ADCs with immune checkpoint inhibitors can counteract antigen heterogeneity by amplifying immune-mediated destruction of tumor cells regardless of antigen expression levels. Similarly, incorporating MDR inhibitors in combination regimens can suppress efflux pump activity, restoring the efficacy of cytotoxic payloads. Advances in linker stability and targeting strategies also offer promising approaches to minimize off-target toxicity and improve therapeutic outcomes.
